# Training curriculum in minimally invasive emergency digestive surgery: 2022 WSES position paper

**DOI:** 10.1186/s13017-023-00476-w

**Published:** 2023-01-27

**Authors:** Nicola de’Angelis, Francesco Marchegiani, Carlo Alberto Schena, Jim Khan, Vanni Agnoletti, Luca Ansaloni, Ana Gabriela Barría Rodríguez, Paolo Pietro Bianchi, Walter Biffl, Francesca Bravi, Graziano Ceccarelli, Marco Ceresoli, Osvaldo Chiara, Mircea Chirica, Lorenzo Cobianchi, Federico Coccolini, Raul Coimbra, Christian Cotsoglou, Mathieu D’Hondt, Dimitris Damaskos, Belinda De Simone, Salomone Di Saverio, Michele Diana, Eloy Espin‐Basany, Stefan Fichtner‐Feigl, Paola Fugazzola, Paschalis Gavriilidis, Caroline Gronnier, Jeffry Kashuk, Andrew W. Kirkpatrick, Michele Ammendola, Ewout A. Kouwenhoven, Alexis Laurent, Ari Leppaniemi, Mickaël Lesurtel, Riccardo Memeo, Marco Milone, Ernest Moore, Nikolaos Pararas, Andrew Peitzmann, Patrick Pessaux, Edoardo Picetti, Manos Pikoulis, Michele Pisano, Frederic Ris, Tyler Robison, Massimo Sartelli, Vishal G. Shelat, Giuseppe Spinoglio, Michael Sugrue, Edward Tan, Ellen Van Eetvelde, Yoram Kluger, Dieter Weber, Fausto Catena

**Affiliations:** 1 Unit of Colorectal and Digestive Surgery, DIGEST Department, Beaujon University Hospital, AP-HP, University of Paris Cité, Clichy, Paris, France; 2 Faculty of Medicine, University of Paris Est, UPEC, Créteil, France; 3 Department of Colorectal Surgery, Queen Alexandra Hospital, University of Portsmouth, Southwick Hill Road, Cosham, Portsmouth, UK; 4 Intensive Care Unit, Bufalini Hospital, Cesena, Italy; 5 Department of General Surgery, IRCCS Policlinico San Matteo Foundation, Pavia, Italy; 6 Department of Surgery, General and Pediatric Surgery, Panama City, Panama; 7 Division of General and Robotic Surgery, Department of Health Sciences, San Paolo Hospital, University of Milan, Milan, Italy; 8 Division of Trauma and Acute Care Surgery, Scripps Memorial Hospital La Jolla, La Jolla, CA USA; 9 Healthcare Administration, Santa Maria Delle Croci Hospital, Ravenna, Italy; 10 General Surgery, San Giovanni Battista Hospital, USL Umbria 2, Foligno, Italy; 11 General and Emergency Surgery, School of Medicine and Surgery, Milano-Bicocca University, Monza, Italy; 12 General Surgery and Trauma Team, ASST Niguarda Milano, University of Milano, Milan, Italy; 13 Department of Digestive Surgery and Liver Transplantation, Michallon Hospital, Grenoble University, Grenoble, France; 14 Department of Clinical, Diagnostic and Pediatric Sciences, University of Pavia, Pavia, Italy; 15 General, Emergency and Trauma Department, Pisa University Hospital, Pisa, Italy; 16 Riverside University Health System Medical Center, Riverside, CA USA; 17 General Surgery Department, ASST-Vimercate, Vimercate, Italy; 18 Department of Digestive and Hepatobiliary/Pancreatic Surgery, Groeninge Hospital, Kortrijk, Belgium; 19 Department of Surgery, Royal Infirmary of Edinburgh, Edinburgh, UK; 20 Department of General and Metabolic Surgery, Poissy and Saint‐Germain‐en‐Laye Hospitals, Poissy, France; 21 Unit of General Surgery, San Benedetto del Tronto Hospital, av5 Asur Marche, San Benedetto del Tronto, Italy; 22 Digestive and Endocrine Surgery, Nouvel Hôpital Civil, University of Strasbourg, Strasbourg, France; 23 IRCAD, Research Institute Against Digestive Cancer, Strasbourg, France; 24 Department of General Surgery, Hospital Valle de Hebron, Universitat Autonoma de Barcelona, Barcelona, Spain; 25 Department of General and Visceral Surgery, Medical Center University of Freiburg, Freiburg, Germany; 26 Department of HBP Surgery, University Hospitals Coventry and Warwickshire NHS Trust, Clifford Bridge Road, Coventry, CV2 2DX UK; 27 Eso-Gastric Surgery Unit, Department of Digestive Surgery, Magellan Center, Bordeaux University Hospital, Pessac, France; 28 Department of Surgery, Sackler School of Medicine, Tel Aviv University, Tel Aviv, Israel; 29 Department of General, Acute Care, Abdominal Wall Reconstruction, and Trauma Surgery, Foothills Medical Centre, Calgary, AB Canada; 30 Digestive Surgery Unit, Health of Science Department, “Magna Graecia” University Medical School, “Mater Domini” Hospital, Catanzaro, Italy; 31 Department of Surgery, Hospital Group Twente ZGT, Almelo, Netherlands; 32 Unit of HPB and Service of General Surgery, Henri Mondor University Hospital, Creteil, France; 33 Department of Gastrointestinal Surgery, University of Helsinki and Helsinki University Hospital, Helsinki, Finland; 34 Department of HPB Surgery and Liver Transplantation, AP-HP Beaujon Hospital, University of Paris Cité, Clichy, France; 35 Unit of Hepato‐Pancreato‐Biliary Surgery, General Regional Hospital “F. Miulli”, Acquaviva delle Fonti, Bari, Italy; 36 Department of Clinical Medicine and Surgery, Federico II” University of Naples, Naples, Italy; 37 Ernest E Moore Shock Trauma Center at Denver Health, University of Colorado, Denver, CO USA; 38 3Rd Department of Surgery, Attikon General Hospital, National and Kapodistrian University of Athens (NKUA), Athens, Greece; 39 University of Pittsburgh School of Medicine, Pittsburgh, PA USA; 40 Visceral and Digestive Surgery, Nouvel Hôpital Civil, University of Strasbourg, Strasbourg, France; 41 Institute for Image‐Guided Surgery, IHU Strasbourg, Strasbourg, France; 42 Institute of Viral and Liver Disease, INSERM U1110, Strasbourg, France; 43 Department of Anesthesia and Intensive Care, Azienda Ospedaliero-Universitaria Parma, Parma, Italy; 44 1St General Surgery Unit, Department of Emergency, ASST Papa Giovanni Hospital Bergamo, Bergamo, Italy; 45 Division of Digestive Surgery, University Hospitals of Geneva and Medical School, Geneva, Switzerland; 46 Minimally Invasive Surgery Fellow, Division of Gastrointestinal and General Surgery, Department of Surgery, Oregon Health and Science University, Portland, OR USA; 47 Department of Surgery, Macerata Hospital, Macerata, Italy; 48 Department of General Surgery, Tan Tock Seng Hospital, Singapore, Singapore; 49 IRCAD Faculty Member Robotic and Colorectal Surgery‐ IRCAD, Strasbourg, France; 50 Department of Surgery, Letterkenny University Hospital, Donegal, Ireland; 51 Department of Surgery, Trauma Surgery, Radboud University Medical Center, Nijmegen, Netherlands; 52 Department of Digestive Surgery, UZ, Brussels, Belgium; 53 Department of General Surgery, Rambam Healthcare Campus, Haifa, Israel; 54 Department of Trauma Surgery, Royal Perth Hospital, Perth, Australia; 55 Department of General and Emergency Surgery, Bufalini Hospital‐Level 1 Trauma Center, Cesena, Italy

**Keywords:** Emergency surgery, Minimally invasive surgery, Robotic surgery, Laparoscopy, Training curriculum in surgery

## Abstract

**Background:**

Minimally invasive surgery (MIS), including laparoscopic and robotic approaches, is widely adopted in elective digestive surgery, but selectively used for surgical emergencies. The present position paper summarizes the available evidence concerning the learning curve to achieve proficiency in emergency MIS and provides five expert opinion statements, which may form the basis for developing standardized curricula and training programs in emergency MIS.

**Methods:**

This position paper was conducted according to the World Society of Emergency Surgery methodology. A steering committee and an international expert panel were involved in the critical appraisal of the literature and the development of the consensus statements.

**Results:**

Thirteen studies regarding the learning curve in emergency MIS were selected. All but one study considered laparoscopic appendectomy. Only one study reported on emergency robotic surgery. In most of the studies, proficiency was achieved after an average of 30 procedures (range: 20–107) depending on the initial surgeon’s experience. High heterogeneity was noted in the way the learning curve was assessed. The experts claim that further studies investigating learning curve processes in emergency MIS are needed. The emergency surgeon curriculum should include a progressive and adequate training based on simulation, supervised clinical practice (proctoring), and surgical fellowships. The results should be evaluated by adopting a credentialing system to ensure quality standards. Surgical proficiency should be maintained with a minimum caseload and constantly evaluated. Moreover, the training process should involve the entire surgical team to facilitate the surgeon’s proficiency.

**Conclusions:**

Limited evidence exists concerning the learning process in laparoscopic and robotic emergency surgery. The proposed statements should be seen as a preliminary guide for the surgical community while stressing the need for further research.

**Supplementary Information:**

The online version contains supplementary material available at 10.1186/s13017-023-00476-w.

## Background

Minimally invasive surgery (MIS), including laparoscopic and robotic approaches, is widely accepted and adopted in elective digestive surgery [1–5]. On the contrary, its application in emergency settings is reserved for a number of selected interventions, mainly approached by laparoscopy [6, 7]. Based on a recent WSES survey conducted amongst 415 surgeons from 67 different countries, laparoscopy was employed for primary emergencies in more than 50% of patients by only 28.7% of participants [8]. For robotic surgery, only 1% of the surgeons declared to use it for more than 25% of the patients with primary emergencies, but, of note, 83.4% of the interviewed surgeons declared to have never used a robotic platform for either elective or emergency surgery [8]. Indeed, the use of robotic surgery for emergency procedures is a matter of debate due to the resources needed (e.g., trained nursing staff, specific equipment availability, dedicated operative theater) and the related costs [8–11]. Moreover, the emergency surgeon willing to perform MIS for surgical emergencies, such as acute cholecystitis, diverticulitis, or appendicitis, must have achieved an adequate proficiency and technical skills in the elective setting to ensure optimal outcomes.

The learning process and skills required for elective digestive MIS are largely documented in the literature [12–15], whereas the corresponding process in emergency surgery has been rarely investigated. This is probably related to a lack of established benchmarks, standards, and goals in the curriculum of the emergency surgeon.

### Project rationale and design

The World Society of Emergency Surgery (WSES) promoted the present position paper to provide a scoping review of the literature describing the learning curve in MIS for emergency digestive/abdominal procedures. The available evidence will constitute the base upon which to develop consensus statements and define future research activities. Under the aegis of the WSES, an organizing committee (composed of Fausto Catena, Nicola de’Angelis, Jim Khan, and Dieter Weber) constituted a Steering Committee in charge of the position paper drafting and invited an International Expert Panel to provide a critical revision of the manuscript.

The process included two steps: (1) perform a review of the available literature describing the learning process and training in minimally invasive emergency digestive surgery using a systematic approach; (2) develop evidence-based statements/recommendations concerning the standards to achieve in MIS for an emergency surgeon.

This position paper was written according to the methodology described by the WSES [16]. As recommended, the GRADE system proposed by the Cochrane Collaboration (https://training.cochrane.org/introduction-grade) was adopted to report the level of evidence. The consensus on the position paper statements was assessed through a web survey (by Google Form) open to all the members of the steering committee and the experts’ panel, as well as the board of governors members of the WSES. The consensus was reached if a statement was associated with ≥ 70% of agreement. Otherwise, the statement was re-discussed by email or videoconference, modified, and resubmitted to the experts’ vote until consensus was reached.

## Literature review

### Methods

#### Review question, selection criteria, and search strategy

The present systematic review of the literature aimed to answer to the following focus question: *what are the training process and learning curve in minimally invasive emergency digestive surgery?*

This review was performed following the Cochrane Collaboration-specific protocol [17] and was reported according to the Preferred Reporting Items for Systematic Reviews and Meta-Analyses (PRISMA) statement [18]. Studies describing the learning curve in general surgery emergencies and urgencies were searched in the following databases up to July 2022: Medline (through PubMed), Embase, and Cochrane Library. A specific research query was used for each database, using the following keywords and MeSH terms: robotic, robotic surgery, robotics, robot-assisted, minimally invasive surgery, minimally invasive surgery, laparoscopy, minimally invasive surgical procedures, laparoscopic surgery, learning curve, learning curves, learning, training.

According to the PICOS format, the following items were used to select the articles retrieved from the literature search:

*P, population:* digestive surgeons in training (residents and fellows) or graduated emergency surgeons.

*I, intervention:* digestive surgery interventions performed using a MIS approach in emergency settings.

*C, comparison:* open surgery, minimally invasive surgery (different technique), or no comparison.

*O, outcomes:* surgeon’s learning curve on any outcome, such as operative time or postoperative outcomes.

*S, study design:* due to the expected paucity of studies on the topic in the literature, all types of study design were considered, except case reports.

The literature search and selection were limited to studies written in English. Articles not discriminating between emergency and elective settings were not included, whereas those reporting mixed series were considered eligible only if the percentage of emergency procedures was ≥ 50%. Studies in which the emergency procedures were performed by several surgeons without a specific analysis were not included. Articles reporting hybrid procedures such as hand-assisted laparoscopic interventions were also excluded. Finally, articles assessing pediatric and transplant procedures were not considered.

The literature search and selection were performed by two independent reviewers (FM, CAS). According to the PRISMA methodology, all records were first merged in a single database; duplicates were removed, and the remaining articles were reviewed for relevance on title and abstract. Records were excluded only when both reviewers classified them as non-pertinent. In case of disagreement, a third reviewer (NdeA) was involved in assessing the admissibility of the study. Finally, the two reviewers performed an independent full-text analysis to finalize the inclusion of the potentially pertinent articles.

#### Data extraction and qualitative synthesis

An electronic spreadsheet was filled with data extracted from the original studies selected during the systematic review. The following items were collected: first author’s name, year of publication, scientific journal name, type of study design, time frame of the study, number of patients/procedures evaluated, pathological state requiring surgical intervention, type of surgical intervention, number of surgeons involved, surgeon experience, operative surgical outcomes, postoperative surgical outcomes, learning curve calculation method, expected learning curve.

The risk of bias of the included studies was assessed according to the MINORS scoring system [19]. The MINORS system attributes a score of 0 if the item is not reported, 1 if the item is reported but inadequate, or 2 if the item is reported and adequate. The global highest score is 16 for non-comparative studies and 24 for comparative studies.

## Results

### Literature search and selection

**Fig. 1:**
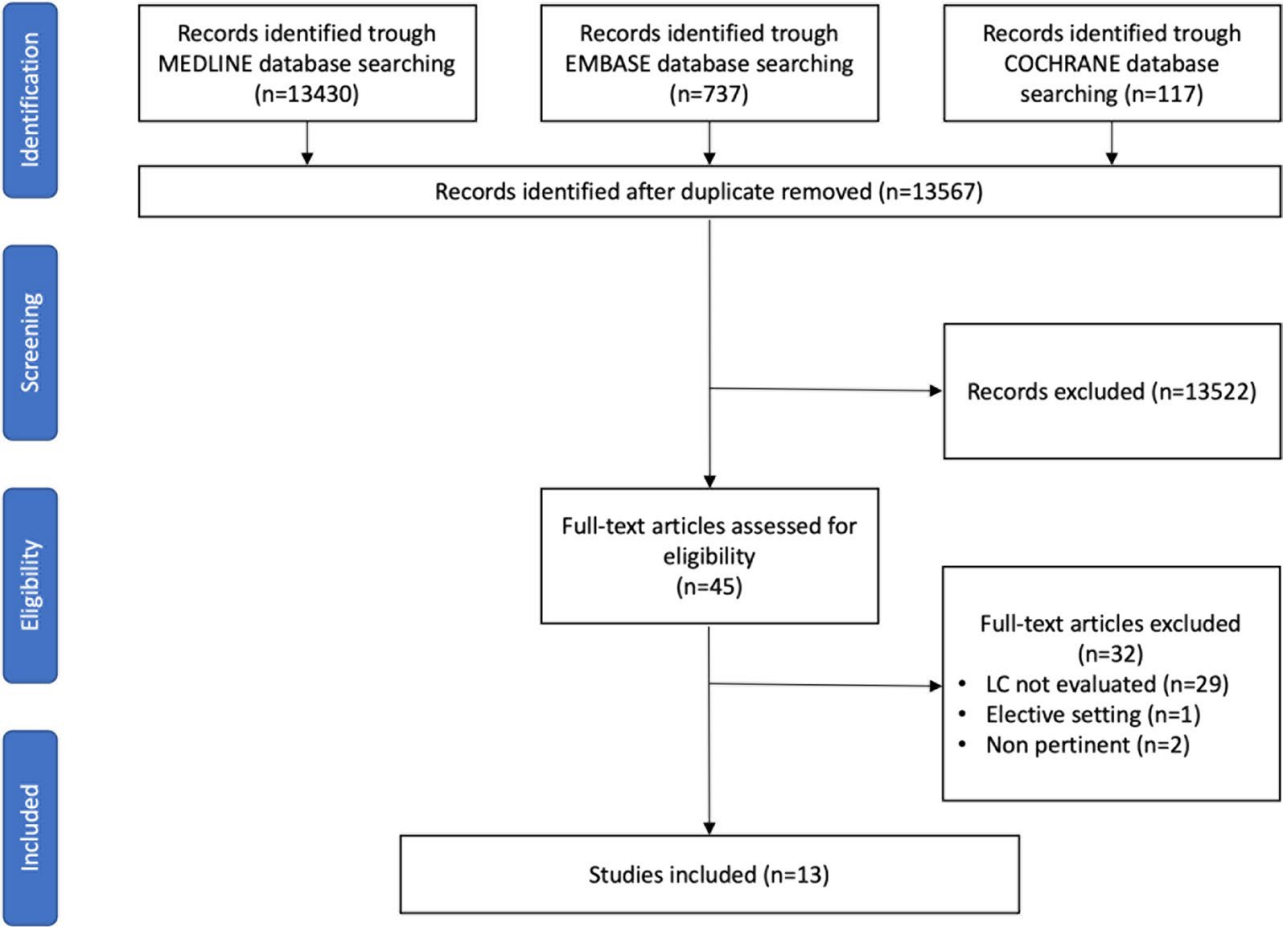
Flowchart of the literature search and selection

Initially, 14,284 records were identified. After duplicate removal, 13,567 articles were screened upon title and abstract. The majority were excluded because non-pertinent to the review question or did not meet all selection criteria. Forty-five articles underwent a full-text evaluation, and 13 studies were finally included in this review (Fig. 1). The list of the excluded articles after full text evaluation is reported in Additional file 1: Table S1.

### Study characteristics

The included studies were published between 2008 and 2022. All were case series carried out in Asia (*n* = 6), Europe (*n* = 5), North America (*n* = 1), or South America (*n* = 1). The characteristics of the included studies are summarized in Table 1. Overall, 4557 minimally invasive emergency procedures were described, of which the great majority (85%) was represented by emergency laparoscopic appendectomy. Only one study reported the learning curve in emergency robotic single-site cholecystectomy [20].

**Table 1:** Studies reporting on learning curve during urgent/emergent minimally invasive surgery

References	Study design	Time period	Surgical emergency	Nb. of pts/procedures	Type of intervention	Surgeons involved	Surgeon experience	Groups calculation and methods	Outcomes (on which LC was evaluated)	Main results	Estimated learning curve
Jaffer et al (2008) [29]	Retrospective observational study - prospectively collected database	May 2005 - November 2006	Appendicitis	40	Laparoscopic appendectomy	1	NR	4 groups (10 pts each) - according to moving average method and CUSUM	OT CR	OT was significantly shorter after 20 cases (p < 0.0001). CR decreased after 20 cases	20 cases are sufficient to gain competences in term of operative time and conversion rate
Kim et al (2010) [27]	Retrospective observational study with subgroup analysis	March 2008 - December 2008	Appendicitis	103 (50 laparoscopic, 53 open)	Laparoscopic and open appendectomy	1	Single 2^nd^ year resident who had performed > 30 open appendectomies (Supervised)	Subgroup analysis for laparoscopic cases: 5 groups (10 pts each) - according to moving average method	OT LOS CR	OT was significantly shorter after 30 cases LOS and CR were comparable	The LC is reached after 30 operations
Lin et al (2010) [21]	Retrospective observational study	January 2002 - December 2007	Appendicitis	240	Laparoscopic appendectomy	6	Residents (FLS certified; trained in basic laparoscopy in wet and simulation laboratories for 2–4 years while assisting in simple laparoscopic surgeries (Supervised)	2 groups (120 pts LC; 120 pts after LC)	OT POC LOS CR	OT was significantly shorter after the LC. (p = 0.005). POC rate was significantly reduced after the LC. (p = 0.04) LOS and CR were not different between the two groups	The LC is reached after 20 cases only for OT
Liao et al (2013) [22]	Retrospective observational study with subgroup analysis	July 2009 - June 2010	Appendicitis	30	Laparoscopic single port appendectomy	1	At least 30 conventional three-port laparoscopic appendectomy	3 groups (10 pts each) - consecutively assigned	OT POC LOS CR TTOI	OT were longest in the first group (P = 0.017). No difference in CR, TTOI, LOS, POC	Significant improvement in OT after the first 10 cases. An experience of 30 cases achieved an OT equivalent to conventional three-port laparoscopic appendectomy First 10 cases had a much steeper downward slope of OT. (–1.5 min/case)
Abdelrahman et al (2016) [31]	Retrospective observational study	August 2007 - August 2014	Appendicitis	/	Laparoscopic and open appendectomy	69	Higher surgical trainees from 3^rd^ to 8^th^ year	According to procedural-based assessment	Procedural-based assessment	Three consultant-validated PBAs at level 4 (competent to perform independently and deal with complications) are reached after 107 cases	The proficiency is reached after 107 cases (median) The number is 35% higher than the number imposed to certify the trainee
Kim et al (2016) [23]	Retrospective observational study	March 2013 - February 2015	Appendicitis	120	Laparoscopic single port appendectomy	1	More than 500 open appendectomy; more than 500 laparoscopic appendectomy	4 groups (30 pts each) - consecutively assigned	OT POC LOS CR TTOI	OT were longest in group A and shortest in group D (P = 0.012) The mean OT was shortened after 30 operations, it was further shortened after 90 operations No difference in POC, LOS, TTOI, CR	Surgical skills can be achieved after 30 operations and more experienced surgical skills after 90 operations
Mán et al (2016) [24]	Retrospective observational study	January 2006 - December 2009	Appendicitis	600	Laparoscopic appendectomy	10	5 residents (2—3 years of surgical experience) Completed a two-week basic laparoscopic skills course and assisted in other laparoscopic procedures (Supervised) 5 consultants (8—9 years of surgical experience) Regularly performed other surgical procedures independently (Supervised)	4 groups (100 pts residents LC; 100 pts consultant LC; 219 residents after LC; 181 consultants after LC) - consecutively assigned	OT POC LOS CR	OT was significantly shorter in both groups (residents and consultants) after the completion of the LC (P < 0.05) The OT was significantly different between the two groups, before and after the completion of the LC (P < 0.05)	The LC is reached after 20 cases both for residents and consultants
Brown et al (2017) [32]	Retrospective observational study	August 2007 - August 2016	Appendicitis	/	Laparoscopic appendectomy	84	Higher surgical trainees from 3rd to 8th year	According to procedural-based assessment	Procedural-based assessment	Three consultant-validated PBAs at level 4 (competent to perform independently and deal with complications) are reached after 95 cases Significant variance was observed in the gradients of all LC related to both the caseload between the first level 3 and the first level 4 PBA (P = 0.001), and between the first and third level 4 PBAs (P < 0.001). Significant variance was also observed in the gradients of all learning curves related to time between the first and third level 4 PBA (P = 0⋅025), but not related to the period between the first level 3 and first level 4 PBA (P = 0.732)	The proficiency is reached after 95 cases (median)
Kim et al (2020) [30]	Retrospective observational study	October 2015 - November 2016	Appendicitis	150	Laparoscopic appendectomy	3	Resident A (1^st^ year 96 surgeries comprising 19 appendectomies and performed 4 laparoscopic appendectomies.) Resident B (2^nd^ year, participated in 272 general surgeries comprising 42 appendectomies and performed 3 laparoscopic appendectomies) Resident C (3^rd^ year, participated in 510 general surgeries comprising 98 appendectomies and performed 4 laparoscopic appendectomies)	(50 pts each resident) - according to moving average method and CUSUM	OT Surgical failure	CUSUM for OT exhibited peaks at the 24th, 18th, and 31st cases for residents A, B, and C, respectively In terms of surgical failure, residents A, B, and C reached steady states after their 35th, 11th, and 16th cases, respectively No significant difference in surgical failure but resident A showed a relatively equal distribution of surgical failure throughout the study period, whereas residents B and C experienced surgical failure earlier on	According to the OR, the LC varies depending on surgical experience ranging from 11 to 35 cases based on a multidimensional analysis
Lee et al (2021) [25]	Retrospective observational study	May 2008 - November 2014	Appendicitis	1948	Laparoscopic single port appendectomy	41	8 surgeons 33 residents (training protocol: at list 10 cases as assistant then first three procedures supervised)	2 groups (483 pts LC; 1465 pts after LC)	OT POC LOS CR HRR Mortality	After a PSM: OT was significantly longer in group 1 than in group 2 (p < 0.001) POC, LOS, CR, HRR and mortality were comparable The rate of incisional hernia tended to be larger in group 1 than in group 2	The LC is reached after 40 cases
Ussia et al (2021) [26]	Retrospective observational study	January 2013 - December 2018	Appendicitis	1173	Laparoscopic appendectomy	73	9 attendings 64 residents (asked to spectate several cases before assisting)	Comparison after PSM: (409 pts attendings 409 pts residents)	OT POC LOS Mortality	After a PSM: LOS was significantly longer in attendings group (p < 0.007) OT, POC and mortality rate were comparable After stratification: OT was significantly reduced only in edematous and suppurative cases as the number of years of training increased CUSUM for OT showed a reduction in OT for attendings at around 300 cases (more than 30 pts/surgeon)	Not specified
Angeramo et al. [28]	Retrospective observational study	June 2000 - December 2019	Postoperative complications in colorectal surgery	132	Various laparoscopic procedures (Lavage and loop ileostomy; resection, redo anastomosis; lavage and drainage; anastomosis takedown; wall repair; bowel repair; adhesiolysis; internal hernia reduction)	3	National board-certified colorectal surgeons	3 groups (50, 52 and 30 pts each) - according to CUSUM analysis (for OT)	OT POC LOS CR Mortality	CR was higher in the first group (P = 0.02) OT was higher in the first group (P = 0.003) Overall postoperative morbidity was lower in the last group (P = 0.01) Major morbidity, mortality and LOS were comparable across the LC	50 re-laparoscopies might be needed to achieve an appropriate LC reducing OT and CR
Kubat et al. [20]	Retrospective observational study with subgroup analysis	May 2012 - August 2013	Acute cholecystitis, biliary pancreatitis, choledocholithiasis, severe chronic cholecystitis, symptomatic cholelithiasis, gallbladder polyposis	150 (76 elective surgery, 74 urgent surgery)	Robotic single port cholecystectomy	1	Experienced minimally invasive surgeon (both in multiport robotic cholecystectomy and in single-incision laparoscopic cholecystectomy)	3 groups (48, 47 and 55 pts each) - according to CUSUM analysis (for OT) Subgroup analysis for urgent cases: 3 groups (35, 34 and 15 pts each)	OT POC LOS CR HRR Mortality	OT was significantly shorter in elective interventions compared with urgent interventions (P < 0.05) LOS was longer in urgent cases (P = 0.003)	The LC is reached after 48 operations, inclusive of urgent and elective cases In the subgroup analysis, the first phase of the CUSUM chart was 25% longer in urgent cases compared to elective cases

*Pts* patients; *LC* learning curve; *NR* not reported; *OT* operative time; *POC* postoperative complications; *LOS* length of stay, *CR* conversion rate; *TTOI* time to oral intake; *CUSUM* cumulative sum; *HRR* hospital re-admission rate; *PSM* propensity score matching; *FLS* fundamentals of laparoscopic surgery

Great heterogeneity in the learning curve assessment was noted; six studies described the chronological distribution in groups and their evaluation [21–26]; one study used the moving average method [27]; two studies used the cumulative sum (CUSUM) analysis [20, 28]; two studies adopted both the moving average method and the CUSUM analysis [29, 30]; and two studies applied the procedural-based assessment [31, 32]. Moreover, six studies evaluated the learning process of resident surgeons [21, 27, 29–32], four of graduated surgeons [20, 22, 23, 28], and three involved both residents and consultants [24–26]. Varying levels of surgical experience were reported in the single studies.

### Qualitative synthesis of the literature

#### Learning curve in emergency laparoscopic appendectomy performed by residents

In 2008, Jaffer et al. [29] published a study reporting the learning curve of a single surgical resident performing 40 cases of laparoscopic appendectomy. By using the moving average method and the CUSUM analysis, the authors demonstrated that the operative time significantly decreased after 20 procedures, indicating the achievement of the learning curve plateau. However, it is noteworthy that the study included a 6% of negative appendicitis, which may lower the difficulty of the emergency procedure.

Similarly, Kim et al. [27] reported in 2010 the experience of a single 2nd year resident dealing with open and laparoscopic appendectomies. The surgeon’s previous experience consisted of 30 open appendectomies supervised by a senior surgeon. According to the moving average method, the subgroup analysis performed on the laparoscopic group demonstrated a significant reduction in the operative time after 30 cases. Length of stay and complication rate were comparable across the consecutive intervention groups.

Lin et al. [21] in 2010 reported the learning process of six residents who performed laparoscopic appendectomy. Based on previous reports, the authors compared the surgical outcomes of the resident’s initial 20 laparoscopic appendectomies (cases performed during the learning curve) with the subsequent 20 cases. A significant reduction in the operative time and rate of postoperative complications was observed with the increasing experience of the residents. The length of hospital stay and conversion to open surgery did not change over time.

In 2016, Abdelrahman et al. [31] published an analysis of the higher surgical trainee’s curriculum from the 3rd to the 8th year. Different from the other studies, the authors adopted the procedural-based assessment (PBA) method, which allows rating the operating surgeon with an increasing level of competence. Levels were defined as follows: level 0 (L0C), insufficient evidence observed to support a judgment; level 1 (L1C), inability to perform the procedure under supervision; level 2 (L2C), ability to perform the procedure under supervision; level 3 (L3C), ability to perform the procedure with minimum supervision; level 4 (L4C), competency to perform the procedure unsupervised and to deal with eventual complications. The authors investigated the achievement of three L4C ratings in 69 residents’ curricula on six index interventions, among which laparoscopic appendectomy. The L4C was achieved in a median of 107 (20–206) appendectomies. The authors compared this number with the national threshold to obtain the surgical certification (80 procedures), highlighting the need for a 35% higher caseload. In 2017, the same research group published an update of the study adopting the same methodology and performing an analysis of the gradient of competence evolution [32]. Concerning appendectomy, the third level 4 PBA proficiency was reached after 95 cases. The study investigated the trajectory of the learning curve and found a significant gradient related to caseload when comparing the first level 3 PBA and the first level 4 PBA and between the first and the third level 4 PBA.

In 2020, Kim et al. [30] published an analysis of 150 laparoscopic appendectomies performed by residents with a growing level of seniority and experience. Three residents, at the first, second or third year of training, performed 50 consecutive laparoscopic appendectomies; no significant differences were shown in operating time (OT) between the three operating residents. Using the moving average method and the CUSUM analysis, the authors reported a decreasing tendency in OT for all residents, with the achievement of peaks between the 18^th^ and 31^st^ cases. When analyzing the need for takeover by the supervisor during the procedure (defined as surgical failure), the steady state on the CUSUM curve was reached between the 11^th^ and 35^th^ cases.

#### Learning curve in emergency laparoscopic appendectomy performed by graduated surgeons

In 2013, Liao et al. [22] reported on the learning curve of single-port laparoscopic appendectomy (SPLA) for noncomplicated appendicitis. The operations were performed by a single surgeon trained in laparoscopic surgery. Thirty SPLA were considered; 3 groups of 10 consecutive SPLA were compared. A significant decrease in OT was observed after 10 cases. Moreover, the study showed that an OT equivalent to a conventional three-port laparoscopic appendectomy was achieved after 30 SPLA cases. No differences were found in the conversion rate, time to resumption of oral intake, length of hospital stay, and postoperative complication rate between the 3 groups of consecutive SPLA.

In 2016, Kim et al. [23] investigated the SPLA learning curve in 120 patients admitted to the emergency department with acute appendicitis. The operations were performed by a single surgeon whose experience was estimated in more than 500 laparoscopic appendectomies and more than 500 conventional open cases. According to the chronological order, 4 groups of 30 consecutive procedures each were compared. A decrease in the OT was observed after 30 cases; no further improvement was reported until 90 operations were completed. No differences were shown between the four groups concerning the rate of postoperative complications, the time to resumption of oral intake, and the length of hospital stay.

#### Learning curve in emergency laparoscopic appendectomy performed by residents and senior surgeons

In 2016, Mán et al. reported a series of 600 laparoscopic appendectomies performed by 5 residents having two or three years of surgical experience and by 5 consultants having eight or nine years of surgical experience [24]. The analyses were performed considering different groups of consecutive interventions: those performed during the learning curve period (the first 100 laparoscopic appendectomies for both residents and consultants) and those performed thereafter (219 cases for residents and 181 for consultants). A significant difference in the OT was reported between residents and consultants both during the learning curve period and after the learning curve completion in favor of consultants. However, a reduction in OT was also observed within each group after the completion of the learning curve.

Lee et al. [25] reported the results of a series of 1948 SPLA performed by 8 attending surgeons and 33 residents. By setting the learning curve achievement threshold at 40 procedures, the authors compared the early cases vs. the subsequent ones. They demonstrated a significant reduction in OT after the completion of the learning curve, whereas the rate of intraoperative and postoperative complications was not different over time.

In 2021, Ussia et al. [26] retrospectively investigated the laparoscopic appendectomies performed in a single surgical unit for six years by nine attending surgeons and 64 residents. The 1173 patients operated on were compared after a propensity score matching, which derived two groups of 409 patients each. The results showed that only the hospitalization was significantly longer in the attendings group compared to residents. When the results were stratified by the inflammatory stage of appendicitis, a significant reduction in OT was present in edematous and suppurative cases. This reduction was progressive according to the year of training, but was not confirmed when including gangrenous appendicitis in the analysis.

#### Learning curve in emergency laparoscopic revisional colorectal surgery performed by graduated surgeons

Angeramo et al. [28] published in 2022 a study describing the learning curve in emergency laparoscopy applied to treat complications of elective laparoscopic colorectal surgery. Between 2000 and 2019, 132 patients underwent a re-operation for postoperative complications by three US board-certified colorectal surgeons. The CUSUM method was used to determine the threshold number of procedures to be performed to reach a stable operative time. Three groups of consecutive procedures (50, 52, and 30 cases respectively) were compared. A higher conversion rate and a longer operative time were observed during the first 50 cases compared to the subsequent cases. Also, the overall morbidity decreased over time, with the lowest rate for the last 30 cases. No differences were reported concerning major morbidity, mortality, and mean length of stay. The authors concluded that 50 laparoscopic interventions should be performed to complete the learning curve.

#### Learning curve in emergency laparoscopic and robotic cholecystectomy performed by graduated surgeons

In 2016, Kubat et al. [20] described the learning curve of robotic single-site cholecystectomy in a case series of the first 150 patients operated on by a single surgeon. The operator was experienced in both multiport robotic cholecystectomy and single-incision laparoscopic cholecystectomy. Only 74 (49.3%) patients underwent an urgent intervention. The surgeon’s learning curve for OT, including both urgent and elective cases, was reached after 48 operations. A subgroup analysis revealed that the initial learning curve for urgent cases was 25% longer than for elective cases. The authors suggested that developing proficiency in elective cases first may aid the adoption of robotic technology in the urgent setting.

### Study quality assessment

The selected studies were judged of poor to moderate quality, with MINORS scores ranging from 8 to 18 [19] (Table 2). There was great heterogeneity in the methods and outcomes used to assess the learning curve. This is one of the main limitations, which hampers any pooled data analyses and claims for caution in the interpretation and generalizability of the results. The type of training received by the residents and consultant surgeons was rarely described (Table 3), with no standardized pre-clinical training curriculum, including simulation in surgery and hands-on on animal models. Only two studies [21, 24] reported the preclinical training process consisting of surgical simulation and training on animal model but the authors do not specify the time dedicated to training.

**Table 2:** MINORS scores assessing the risk of bias in non-randomized studies

	Clearly stated aim	Inclusion of consecutive patients	Prospective data collection	Endpoints appropriate to study aim	Unbiased assessment of study endpoint	Follow-up appropriate to study aim	< 5% Lost to follow up	Prospective calculation of study size	Adequate control group	Contemporary groups	Baseline equivalence of groups	Adequate statistical analyses	TOTAL MINORS SCORE
**Jaffer et al. **[29]	2	2	2	2	0	2	0	0	NA	NA	NA	NA	10/16
**Kim et al. **[27]	2	2	0	2	0	2	0	0	2	2	1	2	15/24
**Lin et al. **[21]	2	2	0	2	0	2	0	0	NA	NA	NA	NA	8/16
**Liao et al. **[22]	2	2	1	2	0	2	0	0	2	1	2	2	16/24
**Abdelrahman et al. **[31]	2	2	1	2	0	2	0	0	NA	NA	NA	NA	9/16
**Kim et al. **[23]	2	2	0	2	0	2	0	0	NA	NA	NA	NA	8/16
**Mán et al. **[24]	2	2	0	2	0	2	0	0	2	2	2	2	16/24
**Brown et al. **[32]	2	2	1	2	0	2	0	0	NA	NA	NA	NA	9/16
**Kim et al. **[30]	2	2	2	2	0	2	0	1	2	2	1	2	18/24
**Lee et al. **[25]	2	2	0	2	0	2	0	0	2	2	1	2	15/24
**Ussia et al. **[26]	2	2	0	2	0	2	0	0	2	2	2	2	16/24
**Angeramo et al. **[28]	2	2	1	2	0	2	0	0	NA	NA	NA	NA	9/16
**Kubat et al. **[20]	2	2	2	2	0	2	0	0	2	2	1	2	17/24

**Table 3:** Characteristics of the training process in urgent/emergent minimally-invasive digestive surgery described in the selected studies

**Variable** **Reference**	**Type(s) of intervention considered**	**Training process**				
		**Use of surgical simulators**	**Training on animal model**	**Proctoring / Supervised surgeries** **(number of procedures)**	**Progressive training in surgical complexity**	**Previous experience in open surgery**
**Jaffer et al. **[29]	Laparoscopic appendectomy	NR	NR	✓ (NR)	NR	NR
**Kim et al. **[27]	Laparoscopic and open appendectomy	NR	NR	✓ (NR)	NR	✓ > 30 open appendectomies
**Lin et al. **[21]	Laparoscopic appendectomy	✓ FLS certification; wet lab and simulation for 2–4 years	NR	✓ (NR)	✓	NR
**Liao et al. **[22]	Laparoscopic single port appendectomy	NR	NR	NR	✓	NR
**Abdelrahman et al. **[31]	Laparoscopic and open appendectomy	NR	NR	✓ (NR)	NR	✓
**Kim et al. **[23]	Laparoscopic single port appendectomy	NR	NR	NR	NR	✓
**Mán et al. **[24]	Laparoscopic appendectomy	✓ Training box for 2 weeks	✓ Live animals for 2 weeks	✓ (NR)	✓	NR
**Brown et al. **[32]	Laparoscopic appendectomy	NR	NR	✓ (NR)	NR	✓
**Kim et al. **[30]	Laparoscopic appendectomy	NR	NR	✓ (NR)	✓	✓
**Lee et al. **[25]	Laparoscopic single port appendectomy	NR	NR	✓ (3)	✓	NR
**Ussia et al. **[26]	Laparoscopic appendectomy	NR	NR	✓ (NR)	✓	NR
**Angeramo et al. **[28]	Various laparoscopic procedures (Lavage and loop ileostomy; resection, redo anastomosis; lavage and drainage; anastomosis takedown; wall repair; bowel repair; adhesiolysis; internal hernia reduction)	NR	NR	✓ (NR)	✓	✓
**Kubat et al. **[20]	Robotic single port cholecystectomy	NR	NR	NR	NR	NR

*NR* not reported in the article

These data highlight the need for further studies to identify and test the effectiveness of specific training programs to be implemented during the residency (and thereafter) until the surgeons reach proficiency in emergency MIS.

### Position statements

Based on the review of the literature presented above, the following position statements (PS) were proposed and voted by the Expert Panel. For each statement, the supporting literature, the evidence level, and consensus’s strength are reported. Most of the experts involved works in a university hospital/academic center (72.2%) or tertiary care center (27.8%). Most of them has been performing MIS for surgical emergencies for 10–20 years (44.4%) or more than > 20 years (22.2%). Only half of them have access to a robotic platform for surgical emergencies, but with difficult accessibility (27.8%) or only during daytime (11.1%). The experts were trained and reached proficiency in MIS in different ways. They all believe that an emergency surgeon should continue to perform a caseload of elective MIS procedures to remain proficient in emergency MIS.

***PS-1. There is a need for further studies assessing with reliable methods the learning curve process of surgeons in the management of the most common emergencies currently approached with minimally invasive surgical techniques, such as appendicitis, cholecystitis, gastro-intestinal perforations, bowel obstruction, and incarcerated herniae.***

The concept of a learning curve has very old origins, described in 1885 by Ebbinghaus as the retention of memorized information [33]. Today, we refer to the process of granting adequate expertise in a given domain [34]. Particularly in surgery, it requires the repetition of a minimum number of procedures to achieve proficiency [13, 35–37]. Despite the fact that this represents the basis of surgical education, there need to be more studies elucidating the process of the surgical learning curve, particularly in the emergency setting [38, 39]. This potentially translates into disparities in medical education between residency programs, countries, and regions [39, 40]. Most of the time, the surgeon who starts to work autonomously at the end of residency has not yet reach full proficiency in all possible domains of emergency surgery. Mackrill et al. [41] demonstrated that a standardized intervention, such as the laparoscopic appendectomy, has different outcomes if performed by registrars or consultants in Australia. As known, the growing experience is accompanied by a progressive amelioration of the surgical outcomes even after the formal learning curve is completed, meaning after approximately 20–30 cases.

Each minimally invasive procedure may be associated with a different learning process, so data about laparoscopic appendectomy cannot be generalized to laparoscopic cholecystectomy or robotic procedures. Furthermore, laparoscopic appendectomy is a procedure with repeated anatomy which does not include some of the skills required in laparoscopy such the suturing task. Differently, laparoscopic cholecystectomy introduces an anatomical variability without offering the suturing task to the trainer. The literature lacks studies investigating even the most common surgical emergencies that are nowadays approached by MIS, such as appendicitis, cholecystitis, gastrointestinal perforations, bowel occlusion, and incarcerated hernias. Only 13 articles were found in the present systematic review, and 85% of the treated cases consisted of a laparoscopic appendectomy.

It should also be considered that the learning process of an elective procedure may be extended to an emergency one, at least concerning the main technical aspects. Goksoy et al. [42] accurately investigated the learning curve in the elective setting of laparoscopic inguinal hernia repair, concluding that the spectrum of difficulty should be extended gradually and after the completion of the learning curve. This is confirmed by two recent series of robotic abdominal hernia repair where a small number of patients were treated for strangulated hernias. The number of emergency cases increased over the time, showing an extension of the surgeon’s indications and confidence [43, 44]. Kubat et al. [20] described the same progression in robotic single-site cholecystectomies, who suggested that the learning curve could be accelerated by first acquiring the skills necessary to complete elective cases. This concept is not new when considering the studies by Stam et al. [45] and Naguib et al. [46] who highlighted that laparoscopic colorectal resections for diverticular disease are technically challenging and more difficult than the resections performed for oncological indications in an elective setting. The study by Miskovic et al., based on an international multicenter analysis of 4852 cases, confirmed that the learning curve in colorectal surgery should include difficult cases like emergency surgery only in the later stages [47].

*Type of recommendation:* Expert opinion.

*Strength of consensus:* 77.8%

***PS-2. To achieve proficiency, surgical proctoring and dedicated surgical fellowships may have a role that deserves further evaluation. Credentialing systems should be developed to ensure quality standards among different training programs.***

While in the elective setting surgical proctorship is adopted, no literature is available concerning surgical proctoring in the emergency setting. This is not surprising considering the impossibility, by definition, of scheduling an emergency procedure. However, adopting new procedures or technologies should always be accompanied by proctoring to assist surgeons at the beginning of their new activity and support them in difficult situations even when the learning curve is completed [48–52]. In the emergency setting, telesurgery and telementoring including telestration, may have a valuable role that should be further evaluated and applied while using MIS techniques [10, 53–56].

There is an increasing trend toward post-residency surgical sub-specialization, which may become necessary to deliver expert care and master advanced surgical technologies that cannot be completely acquired during residency [57]. In the United States, subspecialization, which is pursued on a voluntary basis, is chosen by the majority of general surgeons before starting an independent activity [58]. The importance of fellowship certification has been recognized in several countries with a documented improvement in surgical outcomes [59, 60]. A good training program can reduce the learning time while maximizing the teaching process; even the simple observation of a standardized procedural step can shorten the learning curve [61] and promote proficiency [62–65].

In the field of emergency surgery, fellowships may represent valuable formal training that allows surgeons to achieve the additional experience needed to operate independently in emergency settings, particularly if the surgeon wishes to practice MIS. Another valid option, which emerged in the literature to verify and maintain a good surgical quality level, is the “qualification or credentialing system”. In 2004, the Japan Society for Endoscopic Surgery established a minimally invasive surgical skill qualification system as a strategy to lower the rate of major postoperative complications after minimally invasive gastrointestinal surgery [66]. Despite the existence of several systems to assess the proficiency of residents and trainers, no previous experience was published assessing the safety and efficacy of a surgical procedure. The system accredited less than 50% of the surgeons who applied at the beginning [66] but the effects of this selection, even if highly debated, produced an improvement in surgical outcomes [67–69]. A recent paper published by Mori et al. [70] showed that in the context of acute cholecystitis, the qualified surgeons outperformed their colleagues in terms of 30 and 90-day mortality. Similarly, Biondo et al. showed that surgeon specialization in colorectal surgery was associated with lower morbidity, mortality, and anastomotic dehiscence rate following emergency colorectal resections, compared to the same interventions performed by general surgeons [71].

The impact of sub-specialization may be even more relevant if considering advanced surgical technologies, like robotic surgery, which require additional training and regular application. However, there needs to be more universally accepted quality standards or credentialing systems to qualify a surgeon as expert in robotic surgery. Some certifications have been proposed in certain specific surgical domains, but most of the time by independent stakeholders. Recently, a consensus conference was held to reply to the public health fear of an increased operative risk for patients undergoing robotic surgery [72]. This initiative engaged experts in the field and produced 76 items in three areas: prerequisite education and training qualifications, surgeon’s performance assessment, ongoing monitoring and surveillance. Despite the systematic approach, the consensus was not evidence-based but relied upon the experience of the participants. Nevertheless, it offered an open frame to adopt in various robotic surgery fields as emergency surgery. These models can be useful to delineate the specific curriculum of an emergency surgeon endowed with MIS skills and to verify the maintenance of the acquired competencies [72–74].

Scientific societies, such as the WSES, should take the lead role in developing educational curricula, like the promotion of fellowships in specific sub-specialties of surgery, the development of an accreditation system ensuring the quality standards of the fellowship, the constitution of a board to deliver specialized training certificates to surgeons having proved their scientific and clinical competences and skills through an established procedural volume and case diversity.

*Type of recommendation:* Expert opinion.

*Strength of consensus:* 80.6%

***PS-3. Training with surgical simulation systems and virtual reality should be standardized and continuously implemented to maintain adequate proficiency and acquisition of new skills.***

The educational process in MIS requires the acquisition of technical skills before facing a real clinical scenario. The compelling educational value of simulation in surgery has made simulation a dedicated field of research [75]. Simulation training may be performed in dry and wet labs and by recurring to virtual reality. Evidence-based curricula for teaching laparoscopic appendectomy and cholecystectomy already exist and can be readily implemented in practice [76, 77]. Their impact on clinical outcomes have been demonstrated; for instance, the adoption of a structured proficiency-based robotic training curriculum for robotic inguinal hernia repair focusing on virtual reality simulation, inanimate bio tissue simulation, and live proctorship was able to positively impact the clinical outcomes and hospital costs [78]. Nevertheless, there are logistic and ethical constraints to the widespread of dry and wet labs that will probably lead to an increased use of virtual reality [79].

A meta-analysis published in 2016 by Alaker et al. [80] showed that virtual reality training can improve operative performance compared to others systems such as box trainers or video trainers. However, when comparing expert surgeons, novices and inexperienced operators, the study was not able to capture the category of surgeons who benefitted the most from such training.

Some widely adopted simulation programs are available to teach and train in MIS, but no specific program is available concerning emergency surgery. Interesting preliminary studies are emerging in this field but not related to MIS and with difficult reproducibility [81].

Technical elements are even more important where the technology is the tool to operate on the patient. As the Fundamentals of Laparoscopic Surgery (FLS) emerged as a tool to teach and assess the fundamental knowledge and technical skills required to safely perform basic laparoscopic surgery [82, 83], the Fundamentals of Robotic Surgery (FRS) was conceived to assess and certify the robotic surgery skills [84]. Despite the simplicity of the educational system, it seems undeniable that a proper mastering of the instruments should be the first step to efficiently start the robotic activity. Furthermore, with the advent of new robotic platforms, the assessment of a universal standard could represent a milestone even though each robot shows an interface specificity.

The simulation field is not free from debate. The literature is very specific, and it is difficult to evaluate the results of the single studies when extrapolated from the local context. In addition, cost-effectiveness should be proven, considering the elevated prices of the simulators. However, stakeholders ranging from hospital managers to patients are showing a growing interest in mandatory simulation prior to clinical activity [85].

*Type of recommendation:* Expert opinion.

*Strength of consensus:* 88.9%

***PS-4. A minimum caseload should be guaranteed in the emergency setting to gain and maintain proficiency in MIS.***

The learning process in MIS is longer and more complex than open surgery because it requires fine perceptual and motor abilities [86]. The skills gained during MIS practice will degrade without use, indicating that once acquired, they should be put into practice shortly and performed regularly [87]. Most reports on this topic are not related to experienced surgeons, but it is well accepted that a minimum number of surgical procedures is required in each domain to reach proficiency. Unfortunately, no evidence is available to establish how many cases a surgeon should perform over time to maintain proficiency. Furthermore, the influence of the transversal competencies in different surgical domains, in elective and emergency settings, has never been investigated.

There is evidence that high-volume surgeons have better outcomes than colleagues working in low-volume centers for colorectal [88], esophageal [89], and hepatobiliary [90] minimally invasive interventions. Also for emergency general surgery, data suggest an association between low-volume surgery and a higher risk of postoperative adverse events [91], particularly in frail populations [92, 93]. Nonetheless, it is difficult to generalize these results to MIS in the emergency setting [94].

In some contexts, such as rural areas, the volume of laparoscopic procedures performed by a single surgeon may be insufficient to safely reach proficiency and overcome the learning curve [95], raising a stringent problem of initial training and continuing education of surgeons and surgical teams. Bruns et al. [96] reported that a reorganization of the acute surgical team could benefit the patient and the hospital. A recent survey published by Ceresoli et al. [8] showed that the main factor related to the adoption of laparoscopy in the emergency setting was the surgeon’s personal experience in elective MIS. Coccolini et al. [97] claimed that one of the pillars of emergency general surgery should be the continuous exposure to surgical activity, which mostly consists of elective procedures. The proposed concept would go beyond the “cumulative volume” analysis. Rotations into a daily elective surgical activity could allow the surgeon to reach the proficiency in MIS necessary to face difficult emergent cases. Not only the single procedure investigated but the total amount of procedures performed by the surgeon in different domains should be considered while adopting MIS. Furthermore, an analysis of the weak area of the surgeon’s curriculum could guarantee the adoption of implementing measures in a less demanding setting.

There is a lack of consensus concerning the interaction between different techniques, such as laparoscopy and robotics. Despite the perception that the two techniques are very similar in approach, views and dissection [98], several studies suggested that previous laparoscopic experience has a limited impact on the robotic proficiency [99, 100]. This finding, associated with the shortened learning curve for robotic surgery, should encourage the adoption of this technology to approach technically demanding cases [101, 102].

*Type of recommendation:* Expert opinion.

*Strength of consensus:* 91.7%

***PS-5. Training programs should target the entire surgical team, whose experience is of utmost importance to facilitating surgeons’ proficiency in MIS in emergency settings.***

The success rate of MIS is determined by a multifactorial combination of the surgeon’s experience and skills, the institution’s equipment and organization, and the surgical team’s competence and specialization. Thus, training, proctoring and continuous education should target the operating surgeon and the entire surgical team.

The surgical team is of foremost importance during emergency surgery, as demonstrated in the daily transmission of competence from the surgeons who had completed the learning curve to colleagues in the learning phase [99, 103].

Moreover, the competence of the nursing staff and the anesthesiology team may also significantly impact the surgical outcomes [104].

Interprofessional collaboration is recognized as a potential way to improve professional practice and healthcare outcomes [105]. It becomes mandatory when the adoption of new surgical technologies generates new organizational challenges [106]. In fact, MIS offers a shared view of the surgical field and gives the opportunity to the entire team to understand the procedure and monitor the operation progress, but it needs the establishment of a new interpersonal routine [107]. Laparoscopy is burdened by the physical stress of the surgical team, whereas robotic surgery offers a less physically demanding approach, but introduces a console creating a physical distance of the surgeon from the team, mediating communication through a microphone [108, 109]. In this context and maybe more in an emergency scenario, a team of well-trained people is the key factor for a successful robotic program [110] and should represent the standard also in emergency MIS. Some strategies to ameliorate team work were described, and the emerging solutions to improve technical skills and communication rely on simulation [111–114].

*Type of recommendation:* Expert opinion

*Strength of consensus:* 97.2%

## Research agenda

A research agenda was established considering the limited available evidence concerning the training in MIS in emergency settings:

- Further studies are needed to determine the learning curve of most emergency procedures performed in MIS. The existing key performance indicators in emergency surgery should be considered when determining the outcomes for a learning curve evaluation [115, 116].
- Future studies should include the evaluation of the baseline experience of the involved surgeons, including previous preclinical training and simulation. They should adopt multidimensional indicators of proficiency in the learning curve analysis such as patient-reported outcomes and long-term results. Furthermore, these studies should report the real costs and sustainability of emergency MIS, particularly concerning the adoption of advanced technologies such as robotic surgery.
- A specific registry should be adopted to collect and assess the type of interventions performed, the surgical volume, the outcomes, and the procedure-related parameters (such as oncologic or patient-reported outcomes). These data may allow the evaluation of the real benefits linked to the adoption of MIS in emergency settings.
- Based on the current evidence and derived from the elective surgery literature, a training curriculum should be defined and proposed to the surgical community. Existing models, such as the laparoscopic cholecystectomy-specific assessment tool (LCAT) [117], could be translated to different procedures and they could help standardize the evaluation of the training. Where not available, a structured assessment should be developed, inspired from the available evidence in the specific field. The assessment should be the complete evaluation of the candidate, considering technical and non-technical skills, medical and surgical knowledge, decision appropriateness, and ability to deal with reactions and complications.
- The comprehensive curriculum for surgeons in training should include information related to the hours of preclinical training, the definition of the step-up approach through the training and the number of procedures as assistant as well as operator. Furthermore, the curriculum should include all the available adopted techniques. Although the training pathway may be adapted to geographical and cultural differences, it will be important to promote quality standard achievements during surgical training. The developed training curriculum should be diffused under the supervision of the WSES to the academic providers of education. To ensure educational excellence, the initiatives adopted by Scientific Societies involved in the educational process should be integrated into a common pathway.

## Conclusion

The training curriculum of the emergency surgeon applying MIS remains under investigation. It appears to be poorly standardized and poorly investigated for major surgical emergencies. In this context, the WSES provided this position paper to summarize the available evidence and propose a successful training pathway in emergency MIS.

## Supplementary Information

Additional file1 (DOCX 25 KB)

## Data Availability

**T**he datasets used during the current study are available from the corresponding author on reasonable request.

## Abbreviations

MIS: Minimally invasive surgery
WSES: World Society of Emergency Surgery
PRISMA: Preferred Reporting Items for Systematic Reviews and Meta-Analyses
CUSUM: Cumulative sum
PBA: Procedural-based assessment
OT: Operating time
SPLA: Single-port laparoscopic appendectomy
PS: Position statements
FLS: Fundamentals of laparoscopic surgery
FRS: Fundamentals of robotic surgery
LCAT: Laparoscopic cholecystectomy-specific assessment tool

## References

[1] Yeo HL, Isaacs AJ, Abelson JS, Milsom JW, Sedrakyan A (2016). Comparison of open, laparoscopic, and robotic colectomies using a large national database: outcomes and trends related to surgery center volume. Dis Colon Rectum.

[2] Asbun HJ, Moekotte AL, Vissers FL, Kunzler F, Cipriani F, Alseidi A (2020). The Miami international evidence-based guidelines on minimally invasive pancreas resection. Ann Surg.

[3] Group MSC (2022). Predictors of surgical outcomes of minimally invasive right colectomy: the MERCY study. Int J Colorectal Dis.

[4] Gotohda N, Cherqui D, Geller DA, Abu Hilal M, Berardi G, Ciria R (2022). Expert consensus guidelines: how to safely perform minimally invasive anatomic liver resection. J Hepatobiliary Pancreat Sci.

[5] Akhtar NM, Chen D, Zhao Y, Dane D, Xue Y, Wang W (2020). Postoperative short-term outcomes of minimally invasive versus open esophagectomy for patients with esophageal cancer: an updated systematic review and meta-analysis. Thorac Cancer.

[6] Zhang G, Wu B (2022). Meta-analysis of the clinical efficacy of laparoscopic appendectomy in the treatment of acute appendicitis. World J Emerg Surg.

[7] Athanasiou C, Lockwood S, Markides GA (2017). Systematic review and meta-analysis of laparoscopic versus open appendicectomy in adults with complicated appendicitis: an update of the literature. World J Surg.

[8] Ceresoli M, Pisano M, Abu-Zidan F, Allievi N, Gurusamy K, Biffl WL (2022). Minimally invasive surgery in emergency surgery: a WSES survey. World J Emerg Surg.

[9] Reinisch A, Liese J, Padberg W, Ulrich F (2022). Robotic operations in urgent general surgery: a systematic review. J Robot Surg.

[10] de’ Angelis N, Khan J, Marchegiani F, Bianchi G, Aisoni F, Alberti D (2022). Robotic surgery in emergency setting: 2021 WSES position paper. World J Emerg Surg.

[11] Sorial RK, Ali M, Kaneva P, Fiore JF, Vassiliou M, Fried GM (2020). Modern era surgical outcomes of elective and emergency giant paraesophageal hernia repair at a high-volume referral center. Surg Endosc.

[12] Barrie J, Jayne DG, Wright J, Murray CJ, Collinson FJ, Pavitt SH (2014). Attaining surgical competency and its implications in surgical clinical trial design: a systematic review of the learning curve in laparoscopic and robot-assisted laparoscopic colorectal cancer surgery. Ann Surg Oncol.

[13] Soomro NA, Hashimoto DA, Porteous AJ, Ridley CJA, Marsh WJ, Ditto R (2020). Systematic review of learning curves in robot-assisted surgery. BJS Open.

[14] Chan KS, Wang ZK, Syn N, Goh BKP (2021). Learning curve of laparoscopic and robotic pancreas resections: a systematic review. Surgery.

[15] Chua D, Syn N, Koh YX, Goh BKP (2021). Learning curves in minimally invasive hepatectomy: systematic review and meta-regression analysis. Br J Surg.

[16] Bala M, Kashuk J, Moore EE, Catena F, Leppaniemi A, Ansaloni L (2018). Establishing position papers by the WSES. World J Emerg Surg.

[17] Higgins JPT, Thomas J, Chandler J, Cumpston M, Li T, Page MJ (2019). Cochrane handbook for systematic reviews of interventions.

[18] Moher D, Liberati A, Tetzlaff J, Altman DG (2009). Preferred reporting items for systematic reviews and meta-analyses: the PRISMA statement. BMJ (Clin Res ed).

[19] Slim K, Nini E, Forestier D, Kwiatkowski F, Panis Y, Chipponi J (2003). Methodological index for non-randomized studies (minors): development and validation of a new instrument. ANZ J Surg.

[20] Kubat E, Hansen N, Nguyen H, Wren SM, Eisenberg D (2016). Urgent and elective robotic single-site cholecystectomy: analysis and learning curve of 150 consecutive cases. J Laparoendosc Adv Surg Tech A.

[21] Lin YY, Shabbir A, So JB (2010). Laparoscopic appendectomy by residents: evaluating outcomes and learning curve. Surg Endosc.

[22] Liao YT, Lin TH, Lee PC, Chou TH, Liang JT, Lin MT (2013). Learning curve of single-port laparoscopic appendectomy for noncomplicated acute appendicitis: a preliminary analysis compared with conventional laparoscopic appendectomy. J Laparoendosc Adv Surg Tech A.

[23] Kim Y, Lee W (2016). The learning curve of single-port laparoscopic appendectomy performed by emergent operation. World J Emerg Surg.

[24] Man E, Nemeth T, Geczi T, Simonka Z, Lazar G (2016). Learning curve after rapid introduction of laparoscopic appendectomy: are there any risks in surgical resident participation?. World J Emerg Surg.

[25] Lee GR, Kim JH, Kim CH, Lee YS, Kim JJ (2021). Single-incision laparoscopic appendectomy is a safe procedure for beginners to perform: experience from 1948 cases. Surg Endosc.

[26] Ussia A, Vaccari S, Gallo G, Grossi U, Ussia R, Sartarelli L (2021). Laparoscopic appendectomy as an index procedure for surgical trainees: clinical outcomes and learning curve. Updates Surg.

[27] Kim SY, Hong SG, Roh HR, Park SB, Kim YH, Chae GB (2010). Learning curve for a laparoscopic appendectomy by a surgical trainee. J Korean Soc Coloproctol.

[28] Angeramo CA, Schlottmann F, Laporte M, Bun ME, Rotholtz NA (2022). Re-laparoscopy to treat early complications after colorectal surgery: Is there a learning curve?. Surg Laparosc Endosc Percutan Tech.

[29] Jaffer U, Cameron AE (2008). Laparoscopic appendectomy: a junior trainee’s learning curve. JSLS.

[30] Kim CW, Jeon SY, Paik B, Bong JW, Kim SH, Lee SH (2020). Resident learning curve for laparoscopic appendectomy according to seniority. Ann Coloproctol.

[31] Abdelrahman T, Long J, Egan R, Lewis WG (2016). Operative experience vs. competence: a curriculum concordance and learning curve analysis. J Surg Educ.

[32] Brown C, Abdelrahman T, Patel N, Thomas C, Pollitt MJ, Lewis WG (2017). Operative learning curve trajectory in a cohort of surgical trainees. Br J Surg.

[33] Wozniak R (1999). Introduction to memory: Hermann Ebbinghaus (1885/1913) Classics in the history of psychology.

[34] Grange P, Mulla M (2015). Learning the “learning curve”. Surgery.

[35] Schouten N, Simmermacher RK, van Dalen T, Smakman N, Clevers GJ, Davids PH (2013). Is there an end of the “learning curve” of endoscopic totally extraperitoneal (TEP) hernia repair?. Surg Endosc.

[36] Reitano E, de’ Angelis N, Schembari E, Carra MC, Francone E, Gentilli S (2021). Learning curve for laparoscopic cholecystectomy has not been defined: a systematic review. ANZ J Surg.

[37] Burghgraef TA, Sikkenk DJ, Verheijen PM, Moumni ME, Hompes R, Consten ECJ (2022). The learning curve of laparoscopic, robot-assisted and transanal total mesorectal excisions: a systematic review. Surg Endosc.

[38] Moore SA, Maduka RC, Tung L, Reilly PM, Morris J, Seamon MJ (2019). Training disparities of our future workforce: a survey of trauma fellowship candidates. J Surg Res.

[39] Singh P, Aggarwal R, Darzi A (2014). Review of selected national surgical curricula: quantity is not the sole marker of quality. J Surg Educ.

[40] McKenna DT, Mattar SG (2014). What is wrong with the training of general surgery?. Adv Surg.

[41] Mackrill D, Allison S (2015). Laparoscopic appendicectomy: An operation for all trainees but does the learning curve continue into consultanthood?. ANZ J Surg.

[42] Goksoy B, Azamat IF, Yilmaz G, Sert OZ, Onur E (2021). The learning curve of laparoscopic inguinal hernia repair: a comparison of three inexperienced surgeons. Wideochir Inne Tech Maloinwazyjne.

[43] Kudsi OY, Bou-Ayash N, Gokcal F, Crawford AS, Chung SK, Chudner A (2022). Learning curve of robot-assisted transabdominal preperitoneal (rTAPP) inguinal hernia repair: a cumulative sum (CUSUM) analysis. Surg Endosc.

[44] Kudsi OY, Gokcal F, Bou-Ayash N, Crawford AS (2022). Learning curve of robotic transversus abdominis release in ventral hernia repair: a cumulative sum (CUSUM) analysis. Surg Endosc.

[45] Stam M, Draaisma WA, Pasker P, Consten E, Broeders I (2017). Sigmoid resection for diverticulitis is more difficult than for malignancies. Int J Colorectal Dis.

[46] Naguib N, Masoud AG (2013). Laparoscopic colorectal surgery for diverticular disease is not suitable for the early part of the learning curve. A retrospective cohort study. Int J Surg.

[47] Miskovic D, Ni M, Wyles SM, Tekkis P, Hanna GB (2012). Learning curve and case selection in laparoscopic colorectal surgery: systematic review and international multicenter analysis of 4852 cases. Dis Colon Rectum.

[48] Sachdeva AK (2021). Preceptoring, proctoring, mentoring, and coaching in surgery. J Surg Oncol.

[49] Bilgic E, Hada T, Dubé T, Valanci S, de Azevedo B, Feldman LS (2021). Defining the key skills required to perform advanced laparoscopic procedures: a qualitative descriptive study. Surg Endosc.

[50] Fowler DL (2010). Enabling, implementing, and validating training methods in laparoscopic surgery. World J Surg.

[51] Scott SI, Dalsgaard T, Jepsen JV, von Buchwald C, Andersen SAW (2020). Design and validation of a cross-specialty simulation-based training course in basic robotic surgical skills. Int J Med Robot.

[52] Eardley NJ, Matzel KE, Gomez Ruiz M, Khan JS, Riley SA, Donnelly MT (2020). European Society of coloproctology colorectal robotic surgery training for the trainers course–the first pilot experience. Colorectal Dis.

[53] Bilgic E, Turkdogan S, Watanabe Y, Madani A, Landry T, Lavigne D (2017). Effectiveness of telementoring in surgery compared with on-site mentoring: a systematic review. Surg Innov.

[54] Antoniou SA, Antoniou GA, Franzen J, Bollmann S, Koch OO, Pointner R (2012). A comprehensive review of telementoring applications in laparoscopic general surgery. Surg Endosc.

[55] Gerardo R, Lele P, Sundaram K, Ponsky T (2021). Surgical telementoring: Feasibility, applicability, and how to. J Surg Oncol.

[56] Erridge S, Yeung DKT, Patel HRH, Purkayastha S (2019). Telementoring of surgeons: a systematic review. Surg Innov.

[57] Grover BT, Kothari SN (2016). Fellowship training: need and contributions. Surg Clin North Am.

[58] Kempenich JW, Dent DL (2021). General surgery resident autonomy: truth and myth. Surg Clin North Am.

[59] Johnston MJ, Singh P, Pucher PH, Fitzgerald JE, Aggarwal R, Arora S (2015). Systematic review with meta-analysis of the impact of surgical fellowship training on patient outcomes. Br J Surg.

[60] Kockerling F, Sheen AJ, Berrevoet F, Campanelli G, Cuccurullo D, Fortelny R (2019). The reality of general surgery training and increased complexity of abdominal wall hernia surgery. Hernia.

[61] Kye BH, Kim JG, Cho HM, Kim HJ, Suh YJ, Chun CS (2011). Learning curves in laparoscopic right-sided colon cancer surgery: a comparison of first-generation colorectal surgeon to advance laparoscopically trained surgeon. J Laparoendosc Adv Surg Tech A.

[62] Nosser M, Feldbrugge L, Pratschke J (2021). Minimally invasive liver surgery: the Charite experience. Turk J Surg.

[63] Chang J, Rattner DW (2019). History of minimally invasive surgical oncology. Surg Oncol Clin N Am.

[64] Halls MC, Alseidi A, Berardi G, Cipriani F, Van der Poel M, Davila D (2019). A comparison of the learning curves of laparoscopic liver surgeons in differing stages of the IDEAL paradigm of surgical innovation: standing on the shoulders of pioneers. Ann Surg.

[65] Gkionis IG, Flamourakis ME, Tsagkataki ES, Kaloeidi EI, Spiridakis KG, Kostakis GE (2020). Multidimensional analysis of the learning curve for laparoscopic colorectal surgery in a regional hospital: the implementation of a standardized surgical procedure counterbalances the lack of experience. BMC Surg.

[66] Mori T, Kimura T, Kitajima M (2010). Skill accreditation system for laparoscopic gastroenterologic surgeons in Japan. Minim Invasive Ther Allied Technol.

[67] Kikuchi S, Kagawa T, Kuroda S, Nishizaki M, Takata N, Kuwada K (2021). Accreditation as a qualified surgeon improves surgical outcomes in laparoscopic distal gastrectomy. Surg Today.

[68] Akagi T, Endo H, Inomata M, Yamamoto H, Mori T, Kojima K (2020). Clinical impact of Endoscopic Surgical Skill Qualification System (ESSQS) by Japan Society for Endoscopic Surgery (JSES) for laparoscopic distal gastrectomy and low anterior resection based on the National Clinical database (NCD) registry. Ann Gastroenterol Surg.

[69] Ichikawa N, Homma S, Hida K, Akagi T, Kamada Y, Yamaguchi T (2022). Impact of endoscopic surgical skill qualification on laparoscopic resections for rectal cancer in Japan: the EnSSURE study. Ann Surg Open.

[70] Mori T, Endo H, Misawa T, Yamaguchi S, Sakamoto Y, Inomata M (2022). Involvement of a skill-qualified surgeon favorably influences outcomes of laparoscopic cholecystectomy performed for acute cholecystitis. Surg Endosc.

[71] Biondo S, Kreisler E, Millan M, Fraccalvieri D, Golda T, Frago R (2010). Impact of surgical specialization on emergency colorectal surgery outcomes. Arch Surg.

[72] Stefanidis D, Huffman EM, Collins JW, Martino MA, Satava RM, Levy JS (2022). Expert consensus recommendations for robotic surgery credentialing. Ann Surg.

[73] Petz W, Spinoglio G, Choi GS, Parvaiz A, Santiago C, Marecik S (2016). Structured training and competence assessment in colorectal robotic surgery. Results of a consensus experts round table. Int J Med Robot.

[74] Awad M, Awad F, Carter F, Jervis B, Buzink S, Foster J (2018). Consensus views on the optimum training curriculum for advanced minimally invasive surgery: a delphi study. Int J Surg.

[75] Fritz T, Stachel N, Braun BJ (2019). Evidence in surgical training–a review. Innov Surg Sci.

[76] Sinitsky DM, Fernando B, Potts H, Lykoudis P, Hamilton G, Berlingieri P (2020). Development of a structured virtual reality curriculum for laparoscopic appendicectomy. Am J Surg.

[77] Nayar SK, Musto L, Fernandes R, Bharathan R (2019). Validation of a virtual reality laparoscopic appendicectomy simulator: a novel process using cognitive task analysis. Ir J Med Sci.

[78] Tam V, Borrebach J, Dunn SA, Bellon J, Zeh HJ, Hogg ME (2019). Proficiency-based training and credentialing can improve patient outcomes and decrease cost to a hospital system. Am J Surg.

[79] Sankaranarayanan G, Parker L, De S, Kapadia M, Fichera A (2021). Simulation for colorectal surgery. J Laparoendosc Adv Surg Tech A.

[80] Alaker M, Wynn GR, Arulampalam T (2016). Virtual reality training in laparoscopic surgery: a systematic review & meta-analysis. Int J Surg.

[81] Tadlock MD, Olson EJ, Gasques D, Champagne R, Krzyzaniak MJ, Belverud SA (2022). Mixed reality surgical mentoring of combat casualty care related procedures in a perfused cadaver model: Initial results of a randomized feasibility study. Surgery.

[82] Zendejas B, Ruparel RK, Cook DA (2016). Validity evidence for the Fundamentals of Laparoscopic Surgery (FLS) program as an assessment tool: a systematic review. Surg Endosc.

[83] Cullinan DR, Schill MR, DeClue A, Salles A, Wise PE, Awad MM (2017). Fundamentals of laparoscopic surgery: not only for senior residents. J Surg Educ.

[84] Satava RM, Stefanidis D, Levy JS, Smith R, Martin JR, Monfared S (2020). Proving the effectiveness of the fundamentals of robotic surgery (FRS) skills curriculum: a single-blinded, multispecialty. Multi Inst Randomized Control Trial Ann Surg.

[85] Preshaw J, Siassakos D, James M, Draycott T, Vyas S, Burden C (2019). Patients and hospital managers want laparoscopic simulation training to become mandatory before live operating: a multicentre qualitative study of stakeholder perceptions. BMJ Simul Technol Enhanc Learn.

[86] Spruit EN, Band GP, Hamming JF, Ridderinkhof KR (2014). Optimal training design for procedural motor skills: a review and application to laparoscopic surgery. Psychol Res.

[87] Gallagher AG, Jordan-Black JA, O'Sullivan GC (2012). Prospective, randomized assessment of the acquisition, maintenance, and loss of laparoscopic skills. Ann Surg.

[88] Huo YR, Phan K, Morris DL, Liauw W (2017). Systematic review and a meta-analysis of hospital and surgeon volume/outcome relationships in colorectal cancer surgery. J Gastrointest Oncol.

[89] Derogar M, Sadr-Azodi O, Johar A, Lagergren P, Lagergren J (2013). Hospital and surgeon volume in relation to survival after esophageal cancer surgery in a population-based study. J Clin Oncol.

[90] Franchi E, Donadon M, Torzilli G (2020). Effects of volume on outcome in hepatobiliary surgery: a review with guidelines proposal. Glob Health Med.

[91] Mehta A, Efron DT, Canner JK, Dultz L, Xu T, Jones C (2017). Effect of surgeon and hospital volume on emergency general surgery outcomes. J Am Coll Surg.

[92] Ang D, Sugimoto J, Richards W, Liu H, Kinslow K, McKenney M (2021). Hospital volume of emergency general surgery and its impact on inpatient mortality for geriatric patients: analysis from 3994 hospitals. Am Surg.

[93] Mehta A, Dultz LA, Joseph B, Canner JK, Stevens K, Jones C (2018). Emergency general surgery in geriatric patients: a statewide analysis of surgeon and hospital volume with outcomes. J Trauma Acute Care Surg.

[94] Wohlgemut JM, Ramsay G, Bekheit M, Scott NW, Watson AJM, Jansen JO (2021). Emergency general surgery: impact of hospital and surgeon admission case volume on mortality. J Trauma Acute Care Surg.

[95] Moore J, Pellet A, Hyman N (2016). Laparoscopic Colectomy and the General Surgeon. J Gastrointest Surg.

[96] Bruns BR, Tesoriero RB, Narayan M, O'Meara L, Lauerman MH, Eaton B (2016). Acute care surgery and emergency general surgery: addition by subtraction. J Trauma Acute Care Surg.

[97] Coccolini F, Sartelli M, Kluger Y, Osipov A, Cui Y, Beka SG (2022). The LIFE TRIAD of emergency general surgery. World J Emerg Surg.

[98] Jimenez-Rodriguez RM, Rubio-Dorado-Manzanares M, Diaz-Pavon JM, Reyes-Diaz ML, Vazquez-Monchul JM, Garcia-Cabrera AM (2016). Learning curve in robotic rectal cancer surgery: current state of affairs. Int J Colorectal Dis.

[99] Noh GT, Han M, Hur H, Baik SH, Lee KY, Kim NK (2021). Impact of laparoscopic surgical experience on the learning curve of robotic rectal cancer surgery. Surg Endosc.

[100] Kim MS, Kim WJ, Hyung WJ, Kim HI, Han SU, Kim YW (2021). Comprehensive learning curve of robotic surgery: discovery from a multicenter prospective trial of robotic gastrectomy. Ann Surg.

[101] Leijte E, de Blaauw I, Van Workum F, Rosman C, Botden S (2020). Robot assisted versus laparoscopic suturing learning curve in a simulated setting. Surg Endosc.

[102] Flynn J, Larach JT, Kong JCH, Waters PS, Warrier SK, Heriot A (2021). The learning curve in robotic colorectal surgery compared with laparoscopic colorectal surgery: a systematic review. Colorectal Dis.

[103] Guend H, Widmar M, Patel S, Nash GM, Paty PB, Guillem JG (2017). Developing a robotic colorectal cancer surgery program: understanding institutional and individual learning curves. Surg Endosc.

[104] Hallet J, Jerath A, Turgeon AF, McIsaac DI, Eskander A, Zuckerman J (2021). Association between anesthesiologist volume and short-term outcomes in complex gastrointestinal cancer surgery. JAMA Surg.

[105] Reeves S, Pelone F, Harrison R, Goldman J, Zwarenstein M (2017). Interprofessional collaboration to improve professional practice and healthcare outcomes. Cochrane Database Syst Rev.

[106] Schuessler Z, Scott Stiles A, Mancuso P (2020). Perceptions and experiences of perioperative nurses and nurse anaesthetists in robotic-assisted surgery. J Clin Nurs.

[107] Catchpole K, Bisantz A, Hallbeck MS, Weigl M, Randell R, Kossack M (2019). Human factors in robotic assisted surgery: Lessons from studies 'in the Wild'. Appl Ergon.

[108] Park LS, Pan F, Steffens D, Young J, Hong J (2021). Are surgeons working smarter or harder? A systematic review comparing the physical and mental demands of robotic and laparoscopic or open surgery. World J Surg.

[109] Gillespie BM, Gillespie J, Boorman RJ, Granqvist K, Stranne J, Erichsen-Andersson A (2021). The impact of robotic-assisted surgery on team performance: a systematic mixed studies review. Hum Factors.

[110] Giedelman C, Covas Moschovas M, Bhat S, Brunelle L, Ogaya-Pinies G, Roof S (2021). Establishing a successful robotic surgery program and improving operating room efficiency: literature review and our experience report. J Robot Surg.

[111] Chheang V, Fischer V, Buggenhagen H, Huber T, Huettl F, Kneist W (2020). Toward interprofessional team training for surgeons and anesthesiologists using virtual reality. Int J Comput Assist Radiol Surg.

[112] Lorello GR, Cook DA, Johnson RL, Brydges R (2014). Simulation-based training in anaesthesiology: a systematic review and meta-analysis. Br J Anaesth.

[113] Ballas D, Cesta M, Roulette GD, Rusnak M, Ahmed R (2018). Emergency undocking in robotic surgery: a simulation curriculum. J Vis Exp.

[114] Huser AS, Muller D, Brunkhorst V, Kannisto P, Musch M, Kropfl D (2014). Simulated life-threatening emergency during robot-assisted surgery. J Endourol.

[115] Sugrue M, Maier R, Moore EE, Boermeester M, Catena F, Coccolini F (2017). Proceedings of resources for optimal care of acute care and emergency surgery consensus summit Donegal Ireland. World J Emerg Surg.

[116] Sugrue M, Maier R, Moore EE, Catena F, Coccolini F, Kluger Y (2020). Resources for optimal care of emergency surgery.

[117] Chevallay M, Liot E, Fournier I, Abbassi Z, Peloso A, Hagen ME (2022). Implementation and validation of a competency assessment tool for laparoscopic cholecystectomy. Surg Endosc.

