# Supplementary material for: Training curriculum in minimally invasive emergency digestive surgery: 2022 WSES position paper
Source: World J Emerg Surg. 2023 Jan 27;18:11. doi: 10.1186/s13017-023-00476-w (PMC9883976; doi:10.1186/s13017-023-00476-w)
Supplement: Supplementary file 1 — Additional file1 (DOCX 25 KB) [file 13017_2023_476_MOESM1_ESM.docx]

**Supplemental Table 1. Excluded studies and reason for exclusion.**

| **Year of publication** | **Authors** | **Title** | **Reason for exclusion** |
| --- | --- | --- | --- |
| 2020 | de Almeida Medeiros et al. | Treating incarcerated inguinal hernias with TEP is a viable option for experienced surgeons | LC not reported |
| 1994 | Meinke et al. | What is the learning curve for laparoscopic appendectomy? | LC not reported |
| 1996 | Liu et al. | Factors Affecting Conversion of Laparoscopic Cholecystectomy to Open Surgery | Mixed emergency/elective |
| 1998 | Tarnoff et al. | A Comparison of Laparoscopic and Open Appendectomy | LC not reported |
| 2001 | Katkhouda et al. | Intraabdominal Abscess Rate after Laparoscopic Appendectomy | LC not reported |
| 2002 | Bingener-Casey et al. | Reasons for Conversion From Laparoscopic to Open Cholecystectomy: A 10-Year Review | LC not reported |
| 2004 | Agresta et al. | Laparoscopic Appendectomy in Italy: An Appraisal of 26,863 Cases | LC not reported |
| 2006 | Chiu et al. | Role of Appendectomy in Laparoscopic Training | LC not reported |
| 2007 | Pandey et al. | Laparoscopic appendicectomy: a training model for laparoscopic right hemicolectomy? | LC not reported |
| 2007 | Teoh et al. | Routine early laparoscopic cholecystectomy for acute cholecystitis after conclusion of a randomized controlled trial | LC not reported |
| 2008 | Ali et al. | Recent experience with laparoscopic appendectomy in a Canadian teaching centre | LC not reported |
| 2010 | Chouillard et al. | Single-incision laparoscopic appendectomy for acute appendicitis: a preliminary experience | LC not reported |
| 2010 | Wiseman et al. | Surgical Management of Acute Cholecystitis at a Tertiary Care Center in the Modern Era | LC not reported |
| 2010 | Fowler et al. | Enabling, Implementing, and Validating Training Methods in Laparoscopic Surgery | LC not reported |
| 2011 | Vettoretto et al. | Consensus conference on laparoscopic appendectomy: development of guidelines | LC not reported |
| 2011 | Goel et al. | Single-incision Laparoscopic Appendectomy: Prospective Case Series at a Single Centre in Singapore | LC not reported |
| 2013 | Naguib et al. | Laparoscopic colorectal surgery for diverticular disease is not suitable for the early part of the learning curve. A retrospective cohort study | LC not reported |
| 2013 | Uecker et al. | Comparable Operative Times With and Without Surgery Resident Participation | LC not reported |
| 2013 | Schwartz et al. | Senior Residents as Teaching Assistants During Laparoscopic Cholecystectomy in the 80-Hour Workweek Era: Effect on Biliary Injury and Overall Complication Rates | LC not reported |
| 2015 | Mackrill et al. | Laparoscopic appendicectomy: an operation for all trainees but does the learning curve continue into consultanthood? | LC not reported |
| 2015 | Kim et al. | Laparoscopic Surgery for Perforated Duodenal Ulcer Disease: Analysis of 70 Consecutive Cases From a Single Surgeon | LC not reported |
| 2016 | Suh et al. | Single-Incision Laparoscopic Appendectomy by Surgical Trainees | LC not reported |
| 2016 | Gifford et al. | The effect of residents as teaching assistants on operative time in laparoscopic cholecystectomy | LC not reported |
| 2016 | De Win et al. | An evidence-based laparoscopic simulation curriculum shortens the clinical learning curve and reduces surgical adverse events | LC not reported |
| 2017 | Celentano et al. | Laparoscopic sigmoid resection for diverticulitis is rarely a suitable case for the initial phase of the learning curve | Non pertinent |
| 2017 | Stam et al. | Sigmoid resection for diverticulitis is more difficult than for malignancies | LC not reported |
| 2017 | Sirimanna et al. | Development of a proficiency-based virtual reality simulation training curriculum for laparoscopic appendicectomy | LC not reported |
| 2019 | Jabbar et al. | Laparoscopic Training Opportunities in an Emergency Biliary Service | LC not reported |
| 2020 | Sinitsky et al. | Development of a structured virtual reality curriculum for laparoscopic appendicectomy | Non pertinent |
| 2020 | Amin-Tai et al. | Acquiring Advanced Laparoscopic Colectomy Skills – The Issues | LC not reported |
| 2021 | Pang et al. | Structured Training for Laparoscopic Appendectomy for Residents (STAR Trial)–A Randomized Pilot Study | LC not reported |
| 2021 | Bilgic et al. | Defining the key skills required to perform advanced laparoscopic procedures: a qualitative descriptive study | LC not reported |

LC: learning curve.
